# Association of perioperative bioactive adrenomedullin with early postoperative organ dysfunction after major hepatic resection: An exploratory observational study

**DOI:** 10.1007/s00423-026-04148-6

**Published:** 2026-07-23

**Authors:** Jan Bardenhagen, Courtney Metz, Christine Torrey, Tarik Ghadban, Mara R Goetz, Fiete Gehrisch, Jakob R Izbicki, Asmus Heumann, Thilo Hackert, Valerie Isabel Nottberg

**Affiliations:** Department of General, Visceral and Thoracic Surgery, University Medical Center Hamburg-Eppendorf, Martinistraße 52, 20246 Hamburg, Germany

**Keywords:** Biomarker, Hepatic surgery, Outcome, Perioperative management, Organ dysfunction

## Abstract

**Purpose:**

To explore the association between perioperative bioactive adrenomedullin (bioADM) and early postoperative organ dysfunction after major hepatic resection, using the Sequential Organ Failure Assessment (SOFA) score as a measure of organ dysfunction severity.

**Methods:**

This prospective, observational single-center study included 17 patients undergoing major hepatic resection between January and June 2023. BioADM was measured preoperatively and on postoperative days (POD) 1, 2, 3, and 5. Associations with SOFA were assessed using Spearman’s rank correlation. Secondary exploratory analyses evaluated organ-specific and overall postoperative outcomes.

**Results:**

Perioperative bioADM was associated with postoperative organ dysfunction severity as reflected by SOFA. Correlations were weak at baseline and POD1 but became moderate to strong from POD2 onward, including POD2 (ρ = 0.74, *p* = 0.001), POD3 (ρ = 0.64, *p* = 0.012), and POD5 (ρ = 0.67, *p* = 0.008). Exploratory secondary analyses suggested associations with vasopressor requirement and renal deterioration, particularly dialysis requirement. In contrast, bioADM showed no consistent association with major complications, Clavien-Dindo grade, 30-day Comprehensive Complication Index, or 30-day mortality.

**Conclusion:**

In this small exploratory cohort (*n* = 17), perioperative bioADM was associated with early postoperative organ dysfunction after major hepatic resection, particularly from POD2 onward, but not with broader measures of postoperative morbidity or short-term mortality. Given the limited sample size and low number of events, these findings are hypothesis-generating and require validation in larger prospective cohorts before any conclusions regarding clinical utility can be drawn.

**Supplementary Information:**

The online version contains supplementary material available at https://doi.org/10.1007/s00423-026-04148-6.

## Introduction

Major hepatic resection remains a cornerstone in the treatment of primary and secondary liver malignancies as well as selected benign hepatobiliary conditions. Despite substantial advances in operative technique and perioperative care, these procedures remain associated with substantial postoperative morbidity [1, 2]. Beyond procedure-specific complications, patients undergoing major liver surgery may develop systemic postoperative deterioration characterized by circulatory instability, renal dysfunction, and multiorgan impairment with overall complication-rates of up to 47% [2, 3]. Early identification of patients at risk for such postoperative organ dysfunction remains challenging and clinically important.

In this context, biomarkers may provide additional value by capturing pathophysiological processes that are not fully reflected by conventional clinical parameters alone. Bioactive adrenomedullin (bioADM) is a vasoactive peptide involved in the regulation of endothelial integrity, vascular tone, and microcirculatory homeostasis. Endothelial cells have been shown to actively synthesize and secrete adrenomedullin [4]. Elevated circulating bioADM levels have been associated with endothelial dysfunction, hemodynamic instability, and adverse outcomes in critical illness, particularly in conditions characterized by systemic inflammation and organ failure [5, 6]. These properties make bioADM an attractive candidate biomarker for the perioperative setting, where postoperative systemic stress and organ dysfunction may evolve dynamically over time.

Evidence from non-hepatic settings supports this rationale. Previous studies have linked elevated perioperative bioADM levels to vasopressor requirement, septic complications, and mortality, particularly in critically ill and vascular surgical populations [4, 6–9]. However, data on bioADM in major abdominal surgery remain limited, and its perioperative role in hepatic resection has not been well defined. In particular, it is unclear whether bioADM reflects general postoperative morbidity or rather more specifically tracks evolving postoperative organ dysfunction.

This distinction is especially relevant in hepatic surgery. Postoperative outcome after major liver resection is heterogeneous and ranges from localized surgical complications to systemic deterioration with impaired organ perfusion, inflammatory dysregulation, and progressive multiorgan dysfunction. A biomarker linked to endothelial and circulatory stress may therefore be more informative for postoperative organ dysfunction severity than for overall complication burden alone. Of note, throughout this manuscript ‘organ dysfunction’ refers to systemic dysfunction across multiple organ systems as captured by the SOFA score (e.g., cardiovascular, renal, respiratory, hepatic, coagulation, and neurological), and not specifically to post-hepatectomy liver dysfunction, which is governed by distinct, procedure-specific pathophysiology and was not the focus of this analysis.

The aim of this exploratory analysis was to investigate the association between perioperative bioactive adrenomedullin and early postoperative organ dysfunction following major hepatic resection, using the Sequential Organ Failure Assessment (SOFA) score as a measure of organ dysfunction severity. We hypothesized that perioperative bioADM would be associated with the severity of postoperative organ dysfunction after major hepatic resection, as reflected by SOFA.

## Materials and methods

### Study design and setting

We conducted a prospective, observational, single-center study at the Department of Surgery of the University Medical Center Hamburg-Eppendorf (UKE), Germany. Adult patients scheduled to undergo major hepatic resection were screened for eligibility between January 2023 and June 2023 and enrolled after written informed consent. Biomarker sampling and standardized routine laboratory testing were performed preoperatively and on postoperative days (POD) 1, 2, 3, and 5. Clinical outcomes were assessed during the postoperative course and through 30 days after surgery.

### Patient selection

Eligible patients were aged 18 years or older, able to provide written and oral informed consent, and scheduled to undergo major hepatic resection. For the purpose of this study, major hepatic resection comprised hemihepatectomy, extended hemihepatectomy, trisectorectomy, associating liver partition and portal vein ligation for staged hepatectomy (ALPPS), or anatomical liver resection involving more than two segments. Exclusion criteria were extrahepatic metastatic malignancy, administration of corticosteroids or immunosuppressive agents within four weeks before surgery, severe systemic disease with a life expectancy of less than six weeks, immunosuppression or immune deficiency disorders including human immunodeficiency virus infection, preoperative renal replacement therapy, preoperative septic shock or vasopressor requirement, pregnancy, and incarceration or involuntary institutionalization.

### Enrollment and perioperative sampling

Patients scheduled for elective surgery were screened at least one day before the planned operation. Eight patients initially considered eligible were excluded intraoperatively because of extended unresectable disease. A total of 80 blood samples were obtained at five predefined perioperative time points: 24 h preoperatively (V0), and 24 h (V1), 48 h (V2), 72 h (V3), and 120 h (V5) postoperatively. At each time point, one EDTA tube was collected for point-of-care bioADM testing.

### Biomarker and routine laboratory assessment

Bioactive adrenomedullin (bioADM) was measured using the IB10 analyzer (Sphingotec^®^, Hennigsdorf, Germany) with the corresponding BioADM^®^ assay for quantitative determination in human EDTA whole blood and plasma. Biomarker analysis was performed exclusively for research purposes and was not used for clinical decision-making. In parallel, routine laboratory testing included complete blood count, alanine aminotransferase, aspartate aminotransferase, gamma-glutamyl transferase, alkaline phosphatase, total bilirubin, glutamate dehydrogenase, creatinine, urea, estimated glomerular filtration rate, C-reactive protein, lipase, amylase, procalcitonin, and electrolytes.

### Data collection asnd follow-up

A standardized case report form was used to record demographic characteristics, medical history, comorbidities, perioperative bioADM values, and routine laboratory parameters at all scheduled time points. Follow-up data were extracted from the electronic medical record by qualified physicians and included clinical status, date of discharge, and the occurrence of rehospitalization or death within 30 days after surgery.

### Ethics and reporting standards

The study was approved by the Ethics Committee of the Medical Association of Hamburg (PV3548-1992-BO-ff) and was conducted in accordance with the Declaration of Helsinki. All participants provided written informed consent prior to study inclusion and biomarker sampling. This study was reported in accordance with the Strengthening the Reporting of Observational Studies in Epidemiology (STROBE) statement with the respective data provided in Supplementary Fig. 1.

### Outcomes

The primary exploratory outcome was early postoperative organ dysfunction severity, assessed by the Sequential Organ Failure Assessment (SOFA) score at the predefined perioperative time points [10]. The SOFA score quantifies the degree of dysfunction across six organ systems (respiratory, cardiovascular, hepatic, coagulation, renal, and neurological), with higher scores indicating greater organ dysfunction severity across cardiovascular, renal, respiratory, hepatic, coagulation, and neurological domains. Among the secondary exploratory analyses of organ-specific outcomes, renal dysfunction yielded the most consistent signal (see Results and Discussion), although it represents one of several organ systems captured by SOFA rather than the primary focus of the study. Further secondary exploratory analyses evaluated the association of perioperative bioADM with organ-specific and overall postoperative outcomes, including vasopressor requirement, major complications, postoperative morbidity severity, and short-term mortality. Vasopressor requirement was defined as any vasopressor use during the early postoperative course. Renal dysfunction was assessed by postoperative acute kidney injury (AKIN) and dialysis requirement [11]. The AKIN classification stages acute kidney injury (stage 1–3) based on the rise in serum creatinine and/or reduction in urine output relative to baseline. AKIN was analyzed both as a binary outcome and, where applicable, by stage. Dialysis requirement was defined as the need for postoperative renal replacement therapy within 30 days after surgery or, if earlier, during the index hospitalization. Overall postoperative morbidity was additionally characterized by major complications, defined as Clavien-Dindo grade IIIa or higher, the highest Clavien-Dindo grade within 30 days after surgery, and the 30-day Comprehensive Complication Index (CCI) [12, 13]. Short-term mortality was defined as all-cause mortality within 30 days after surgery. Given the exploratory design and limited sample size, all secondary analyses were considered hypothesis-generating.

### Statistical analysis

Continuous variables were summarized as median with interquartile range (IQR) or mean with standard deviation (SD), as appropriate, and categorical variables as counts with percentages. Associations between perioperative bioADM levels and postoperative organ dysfunction severity, measured by the SOFA score, were analyzed using Spearman’s rank correlation coefficient (ρ) with 95% confidence intervals (CIs). Secondary exploratory analyses of organ-specific and overall postoperative outcomes were performed using ROC curves with corresponding areas under the curve (AUCs), univariable logistic regression for selected binary outcomes, Mann-Whitney U tests for group comparisons, and Spearman’s rank correlation for ordinal outcomes. BioADM values at the assay’s lower reportable limit of 44.9 pg/mL were retained as recorded numeric values and were not modeled as left-censored observations. Missing data were handled by available-case analysis without imputation. Given the exploratory nature of the study, no formal sample size calculation or adjustment for multiple testing was performed, and secondary analyses were considered hypothesis-generating. Statistical analyses were performed using R version 4.5.3 and Python version 3.13.0.

## Results

Of 25 patients screened for eligibility, 17 fulfilled all inclusion criteria and were enrolled, while 8 were excluded intraoperatively because of extended unresectable disease (Supplementary Fig. 1). Seventeen patients undergoing major hepatic resection were included in this exploratory analysis. Median age was 64 years, and 11 patients (64.7%) were male. The most frequent underlying diagnosis was cholangiocarcinoma (8/17, 47.1%), followed by metastatic liver disease (3/17, 17.6%). Standard hemihepatectomy was the most common procedure (11/17, 64.7%), followed by extended hemihepatectomy (6/17, 35.3%). Most procedures were performed via an open approach (12/17, 70.6%), whereas 4 procedures (23.5%) were robotic and 1 (5.9%) laparoscopic. The baseline demographic and perioperative characteristics of the study cohort are summarized in Table 1.

**Table 1:** Baseline and perioperative characteristics of the study cohort

Patients, *n*	17
Age, years, median (range)	64 (41–84)
Sex, n (%)	
Female	6 (35.3)
Male	11 (64.7)
Underlying disease, n (%)	
Cholangiocarcinoma	8 (47.1)
Hepatocellular carcinoma	1 (5.9)
Metastatic liver disease	3 (17.6)
Other malignancy	1 (5.9)
Polycystic liver and kidney disease	1 (5.9)
Benign condition	1 (5.9)
Post-surgical complication	1 (5.9)
Miscellaneous lesion	1 (5.9)
Type of hepatic resection, n (%)	
Extended hemihepatectomy	6 (35.3)
Standard hemihepatectomy	11 (64.7)
Surgical approach, n (%)	
Open	12 (70.6)
Laparoscopic	1 (5.9)
Robotic	4 (23.5)

### Descriptive perioperative bioADM profile

Descriptive analysis of perioperative bioADM concentrations showed that median values remained close to the assay’s lower reportable limit across all predefined time points, while the overall range widened at later postoperative time points (Table 2). Due to early discharge the number of available observations decreased to 15 on POD 3 and to 14 on POD 5 respectively.

**Table 2:** Perioperative bioactive adrenomedullin (bioADM) concentrations at the sampling time points. A substantial proportion of measurements clustered at the assay’s lower reportable limit of 44.9 pg/mL across all time points, particularly at baseline and during the early postoperative course

Time point	*n*	Median (IQR), pg/mL	Min–Max, pg/mL	At lower reportable limit (44.9 pg/mL), *n* (%)
Baseline	17	44.9 (44.9–44.9)	44.9–67.9	16 (94.1)
POD1	17	44.9 (44.9–45.3)	44.9–67.3	12 (70.6)
POD2	17	44.9 (44.9–44.9)	44.9–80.7	13 (76.5)
POD3	15	44.9 (44.9–45.6)	44.9–136.2	11 (73.3)
POD5	14	44.9 (44.9–57.0)	44.9–202.6	9 (64.3)

### Primary exploratory analysis: bioADM and postoperative organ dysfunction

**Fig. 1:**
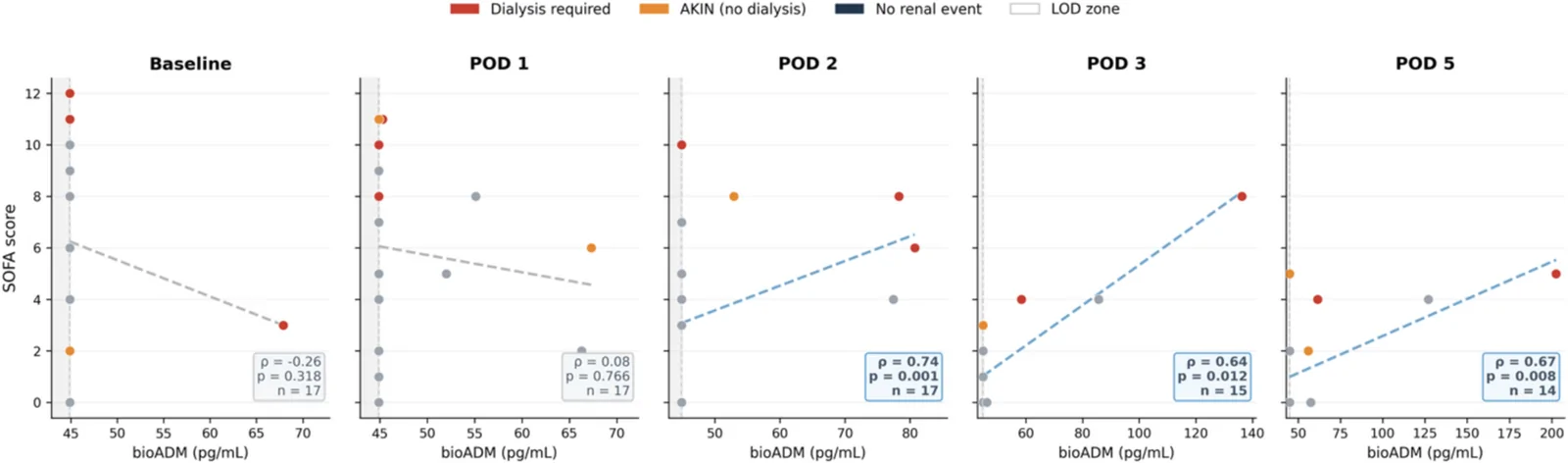
Association between perioperative bioADM concentrations and postoperative organ dysfunction severity Scatterplots showing the relationship between bioactive adrenomedullin (bioADM) concentrations and SOFA score at baseline and on postoperative days (POD) 1, 2, 3, and 5. The shaded area indicates concentrations at or below the assay’s lower reportable limit of 44.9 pg/mL. Correlations were weak at baseline and POD1, but became more pronounced from POD2 onward, supporting a time-dependent association between bioADM and postoperative organ dysfunction.

In the primary exploratory analysis, perioperative bioADM was associated with postoperative organ dysfunction severity as reflected by SOFA. Correlations were weak at baseline and on POD1, but became moderate to strong from POD2 onward. In particular, bioADM correlated with SOFA at POD2 (ρ = 0.74, *p* = 0.001), POD3 (ρ = 0.64, *p* = 0.012), and POD5 (ρ = 0.67, *p* = 0.008) (Fig. 1). Overall, higher bioADM levels paralleled increasing postoperative organ dysfunction during the early postoperative course.

### Secondary exploratory analyses: organ-specific postoperative outcomes

Exploratory analyses showed an association between higher bioADM levels and vasopressor requirement during the early postoperative course (*p* = 0.0356). For postoperative acute kidney injury, patients with AKIN had higher bioADM levels at POD2 than those without AKIN (Mann-Whitney *p* = 0.019; AUC 0.714, 95% CI 0.389–0.976). BioADM was positively associated with AKIN severity at baseline (ρ = 0.657, *p* = 0.004) and POD2 (ρ = 0.691, *p* = 0.004). At POD3, the corresponding analyses yielded *p* = 0.084 for the group comparison and ρ = 0.452 (*p* = 0.096) for the association with AKIN severity. No consistent associations were observed at POD1 or POD5.

**Fig. 2:**
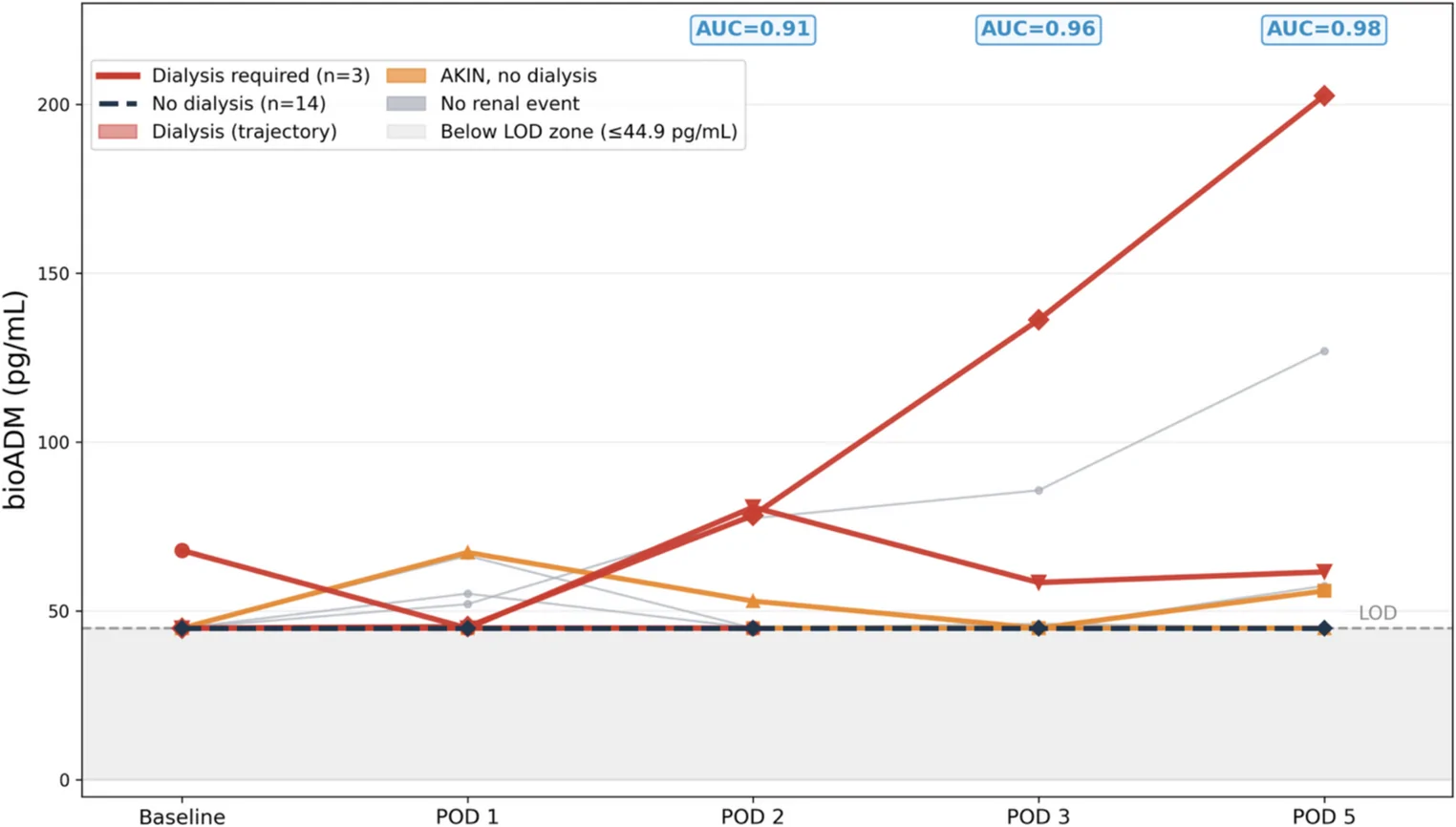
Individual perioperative bioADM trajectories stratified by renal outcome. Individual perioperative bioactive adrenomedullin (bioADM) concentrations at baseline and on postoperative days (POD) 1, 2, 3, and 5, stratified by renal outcome. Patients were grouped as requiring postoperative dialysis, developing AKIN without dialysis, or having no renal event. The shaded area indicates concentrations at or below the assay’s lower reportable limit of 44.9 pg/mL. AUC values shown for POD2, POD3, and POD5 correspond to the discriminatory performance of bioADM for postoperative dialysis requirement at the respective time points

For postoperative dialysis requirement (*n* = 3; indication individually determined based on the clinical presentation, comprising refractory metabolic acidosis with hyperkalemia in two patients and volume overload in the context of pre-existing chronic kidney disease in one patient), discriminatory performance of bioADM increased over time, with AUCs of 0.91 at POD2 (95% CI, 0.73–1.00), 0.96 at POD3 (95% CI, 0.88–1.00), and 0.98 at POD5 (95% CI, 0.92–1.00). Individual perioperative bioADM trajectories stratified by renal outcome are shown in Fig. 2. ROC curves for postoperative dialysis requirement are shown in Supplementary Fig. 2.

Postoperative clinical outcomes of the study cohort are summarized in Table 3. Vasopressor requirement, ICU treatment, renal dysfunction, SOFA scores, complication severity, and 30-day mortality illustrate the substantial postoperative morbidity burden in this cohort and provide the clinical context for the exploratory association analyses reported below.

**Table 3:** Postoperative outcomes during the early postoperative course and within 30 days after surgery. Major complications were defined as Clavien-Dindo grade IIIa or higher. * Percentages based on available measurements at POD5 (*n* = 14). ** Percentages based on available measurements at POD3 (*n* = 15). *** Percentages based on available measurements at POD5 (*n* = 15)

Outcome		Value
Vasopressor support, *n* (%)		
POD0		12 (70.6)
POD1		13 (76.5)
POD2		4 (23.5)
POD3		2 (11.8)
POD5*		1 (7.1)
Dialysis requirement, n (%)		
Any postoperative dialysis		3 (17.6)
POD0		1 (5.9)
POD1		2 (11.8)
POD2		3 (17.6)
POD3		3 (17.6)
POD5*		2 (14.3)
Postoperative ICU treatment, n (%)		
Overall		15 (88.2)
POD0		11 (64.7)
POD1		14 (82.4)
POD2		9 (52.9)
POD3		2 (11.8)
POD5*		3 (21.4)
Acute kidney injury (AKIN), n (%)		
Any stage overall		4 (23.5)
POD0		2 (11.8)
POD1		3 (17.6)
POD2		3 (17.6)
POD3**		3 (18.8)
POD5***		3 (20.0)
SOFA score postoperatively, median (IQR)		
POD1		5 (4–8)
POD2		4 (0–6)
POD3		1 (0–3)
POD5		0 (0–3.5)
Postoperative complications, n (%)		
Any complication (Clavien-Dindo > 0)		14 (82.4)
Major complications (Clavien-Dindo ≥IIIa)		11 (64.7)
Highest Clavien-Dindo grade within 30 days, n (%)		
No complication		3 (17.6)
Grade I		1 (5.9)
Grade II		2 (11.8)
Grade IIIa		3 (17.6)
Grade IIIb		4 (23.5)
Grade IV		3 (17.6)
Grade V		1 (5.9)
30-day Comprehensive Complication Index, median (IQR)		39.5 (20.9–48.1)
30-day mortality, n (%)		1 (5.9)

### Additional exploratory analyses: overall postoperative morbidity and mortality

In contrast to the findings for postoperative organ dysfunction and renal outcomes, bioADM showed no consistent association with broader postoperative morbidity measures. For major complications, baseline discrimination was limited (AUC 0.562, 95% CI, 0.247–0.851), and univariable logistic regression showed an OR of 1.19 per 1-SD increase in bioADM (95% CI, 0.24–5.88; *p* = 0.833). Similar findings were observed across postoperative time points.

BioADM was also not associated with postoperative morbidity severity as assessed by Clavien-Dindo grade or the 30-day Comprehensive Complication Index. At baseline, the correlation with Clavien-Dindo grade was ρ = 0.027 (*p* = 0.917), and the correlation with 30-day CCI was ρ=−0.060 (*p* = 0.828). Corresponding analyses at POD1, POD2, POD3, and POD5 did not show consistent associations. Analyses of 30-day mortality were limited by the limited number of events (*n* = 1).

### Exploratory comparison with creatinine

In exploratory comparisons with routine renal markers, serum creatinine showed higher discriminatory performance than bioADM for postoperative AKIN at most time points, with AUCs of 0.79 at baseline, 1.00 at POD1, 0.91 at POD2, 0.94 at POD3, and 1.00 at POD5. The corresponding AUCs for bioADM were 0.63, 0.63, 0.85, 0.78, and 0.76. For postoperative dialysis requirement, creatinine yielded AUCs of 0.76 at baseline, 0.98 at POD1, 0.83 at POD2, 0.87 at POD3, and 0.95 at POD5, whereas the corresponding AUCs for bioADM were 0.67, 0.48, 0.81, 0.96, and 0.96.

### Discussion

In this exploratory prospective cohort of patients undergoing major hepatic resection, perioperative bioactive adrenomedullin (bioADM) was associated with the severity of early postoperative organ dysfunction, as reflected by SOFA, but not with broader measures of postoperative morbidity or short-term mortality. The most consistent signal emerged from POD2 onward, when higher bioADM levels correlated with increasing SOFA scores and, in exploratory organ-specific analyses, with renal deterioration, particularly dialysis requirement. Taken together, these findings suggest that, in the setting of major hepatic surgery, bioADM may function less as a general predictor of surgical complications and more as a dynamic marker of evolving postoperative organ dysfunction.

This distinction is clinically relevant. Hepatic resection is associated with a broad spectrum of postoperative adverse events, ranging from procedure-specific complications such as bile leakage or bleeding to systemic deterioration characterized by circulatory instability, renal dysfunction, and multiorgan impairment. Our data suggest that bioADM is more closely linked to the latter phenotype. This interpretation is biologically plausible, given the established role of adrenomedullin in endothelial integrity, vascular tone regulation, and microcirculatory homeostasis. In this context, the observed correlation between bioADM and SOFA supports the concept that elevated perioperative bioADM reflects postoperative systemic stress and organ dysfunction rather than overall complication burden per se. This view is further supported by previous studies linking bioADM to renal dysfunction, SOFA score, tumor biology and progression in malignant disease, prognosis in COVID-19, endothelial leakage after pediatric open cardiac surgery, and post-implant right heart failure in LVAD patients, suggesting that endothelial and vascular dysfunction constitute central pathophysiological mechanisms underlying bioADM release across different clinical settings [14–17]. 

The temporal pattern of association further reinforces this interpretation. BioADM showed only weak correlations with SOFA at baseline and POD1, whereas the association became moderate to strong from POD2 onward. This may indicate that bioADM is less informative as a purely preoperative risk marker in this setting and more relevant as a dynamic postoperative marker that tracks the evolution of organ dysfunction over time. Such a time-dependent profile is consistent with the notion that endothelial stress and postoperative circulatory dysregulation become clinically more apparent only after the initial perioperative phase. From a biomarker perspective, this supports the rationale for serial rather than single-time-point measurement.

The exploratory analyses of organ-specific postoperative outcomes yielded the clearest secondary signals in the renal domain. BioADM showed associations with AKIN severity, particularly at baseline and POD2, and its discriminatory performance for dialysis requirement increased markedly from POD2 onward. Although the small number of events precludes firm conclusions, this pattern is noteworthy. It is also consistent with findings from other clinical settings: in critically ill patients with COVID-19, bioADM was associated with early acute kidney injury and predicted the need for renal replacement therapy, including in multivariable analysis [18, 19]. Renal dysfunction after major hepatic surgery is often multifactorial and may reflect hemodynamic instability, endothelial dysfunction, systemic inflammation, venous congestion, and impaired organ perfusion. The observed association between rising postoperative bioADM and later dialysis requirement is therefore consistent with the broader pathophysiological framework suggested by the SOFA findings. Exploratory analyses also suggested a possible association with vasopressor requirement during the early postoperative course, further supporting a link to systemic circulatory dysfunction, although this finding should be interpreted cautiously. Rather than representing an isolated renal biomarker, bioADM may therefore reflect a systemic trajectory of postoperative decompensation in which renal dysfunction constitutes one particularly clinically relevant manifestation.

At the same time, our findings do not support the use of bioADM as a general prognostic marker for overall postoperative morbidity after hepatic resection. While bioADM has been linked to adverse outcomes such as postoperative mortality in cardiac and vascular surgery, in the present study it did not meaningfully discriminate patients with versus without major complications, nor did it correlate with Clavien-Dindo grade, 30-day CCI, or short-term mortality [9, 14]. These negative findings are important because they help define the likely scope of the biomarker more precisely. In this cohort, bioADM was not a broad marker of “any adverse outcome,” but instead appeared to be specifically linked to postoperative organ dysfunction patterns. This narrower interpretation is more consistent with the observed data and likely more useful clinically than an overly broad prognostic claim in the context of hepatic surgery.

The exploratory comparison with creatinine should be interpreted with particular caution. Conventional renal markers, especially creatinine, showed stronger very-early discrimination for postoperative AKIN in this cohort, particularly from POD1 onward. However, given the small sample size and sparse event counts, the present study was not designed to, and cannot, determine whether bioADM provides incremental predictive value beyond creatinine. Rather than suggesting a lack of added value, our findings indicate that bioADM may provide complementary information by reflecting endothelial and circulatory stress, particularly once postoperative organ dysfunction is evolving. This interpretation is supported by previous studies in other clinical settings, in which bioADM provided prognostic information beyond routinely available parameters, including creatinine [20]. Larger comparative studies are therefore needed to determine whether serial bioADM measurements improve risk stratification beyond conventional renal markers in the perioperative setting.

Beyond its role as a biomarker, bioADM has also attracted interest as a potential therapeutic target. This translational perspective is particularly relevant given the growing evidence linking bioADM to systemic inflammatory and septic courses and is conceptually consistent with the organ dysfunction patterns observed in the present study. Adrecizumab, a monoclonal antibody targeting adrenomedullin, has shown favorable safety and tolerability in phase II studies in patients with sepsis [21]. However, randomized controlled trials in patients with cardiogenic shock and elevated bioADM levels have not yet demonstrated a significant clinical benefit [22]. Accordingly, the therapeutic potential of bioADM-targeted strategies remains uncertain and warrants further investigation, particularly in surgical settings, where endothelial dysfunction and postoperative systemic deterioration may represent a distinct pathophysiological context.

The findings of this study should be interpreted in light of several limitations. First, the cohort was small, and rare outcomes such as dialysis and 30-day mortality occurred only in very few patients, resulting in wide confidence intervals and limited precision of effect estimates. Second, the study was exploratory and not powered for confirmatory analyses across multiple clinical endpoints. No adjustment for multiple testing was performed, and all secondary analyses should therefore be regarded as hypothesis-generating. Third, repeated clustering of bioADM values around the lower reportable limit may have reduced the discriminatory granularity of some analyses, particularly threshold-based or rank-based comparisons. Fourth, the cohort was clinically heterogeneous with regard to underlying disease and type of hepatic resection, which may have introduced additional variability. Finally, this was a single-center study, limiting generalizability beyond the local perioperative setting.

Despite these limitations, the study has several strengths. It used a prospective design with standardized serial biomarker measurements at clinically meaningful perioperative time points and focused on a well-defined population undergoing major hepatic surgery. This enabled us to examine not only baseline values but also postoperative biomarker trajectories, which proved particularly informative for the association with organ dysfunction. In addition, the analysis deliberately moved beyond broad complication endpoints and instead explored clinically relevant domains of postoperative deterioration, thereby allowing a more precise characterization of where bioADM may and may not be informative.

From a clinical and translational perspective, the present hypothesis-generating data support further evaluation of bioADM as a dynamic marker of postoperative organ dysfunction after major hepatic resection; however, given the small sample size and the small number of events for key outcomes such as dialysis and mortality, these findings should not be interpreted as evidence of clinical utility at this stage. Future studies should validate these findings in larger and more homogeneous cohorts, ideally with prespecified longitudinal analyses and multivariable adjustment for operative complexity, baseline comorbidity, and postoperative hemodynamic status. In particular, it would be valuable to assess whether serial bioADM measurements improve risk stratification for clinically relevant postoperative decompensation beyond established markers and routine clinical assessment. The most promising application appears to be the identification of patients with evolving systemic dysfunction, especially those on a trajectory toward renal replacement therapy, rather than the prediction of general surgical morbidity.

## Conclusion

In this exploratory cohort of patients undergoing major hepatic resection, perioperative bioactive adrenomedullin was associated with early postoperative organ dysfunction as reflected by SOFA, particularly from postoperative day 2 onward. By contrast, bioADM was not consistently associated with broader measures of postoperative morbidity or short-term mortality. These findings suggest that bioADM may be more useful as a dynamic biomarker of postoperative systemic dysfunction than as a general predictor of surgical complications and support further validation in larger prospective cohorts.

## Electronic Supplementary Material

Below is the link to the electronic supplementary material.

Supplementary Material 1

## Data Availability

The datasets generated and analyzed during the current study are not publicly available due to patient privacy restrictions but are available from the corresponding author upon reasonable request.
