# Supplementary material for: Association of perioperative bioactive adrenomedullin with early postoperative organ dysfunction after major hepatic resection: An exploratory observational study
Source: Langenbecks Arch Surg. 2026 Jul 23;411(1):199. doi: 10.1007/s00423-026-04148-6 (PMC13400470; doi:10.1007/s00423-026-04148-6)
Supplement: Supplementary file 1 — Supplementary Material 1 [file 423_2026_4148_MOESM1_ESM.docx]

**Supplemental Data**


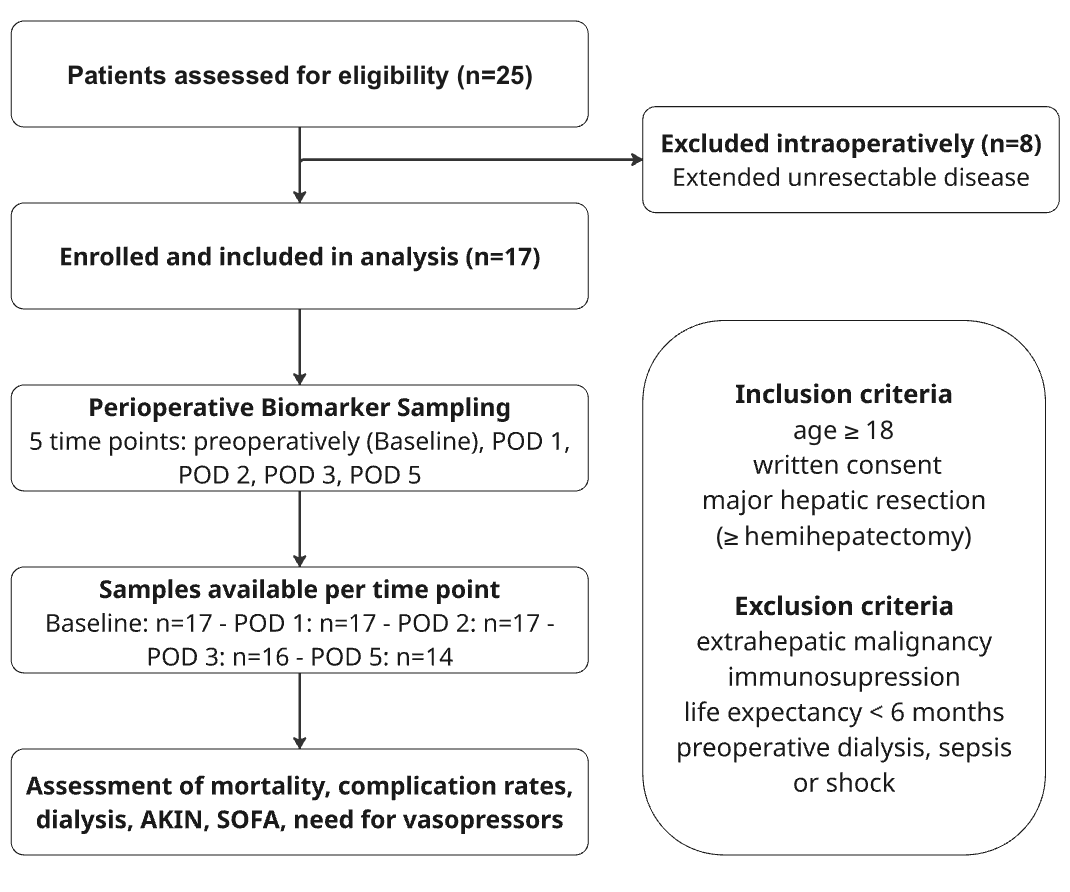


***Supplementary Fig. 1*** *Study Flow Diagram*

Of 25 patients screened for eligibility, 8 were excluded intraoperatively due to extended unresectable disease. A total of 17 patients undergoing major hepatic resection were enrolled and included in the final analysis. Perioperative biomarker sampling was performed at five predefined time points (baseline, POD1, POD2, POD3, and POD5), yielding 80 blood samples in total. The respective inclusion and exclusion criteria are listed on the bottom right.


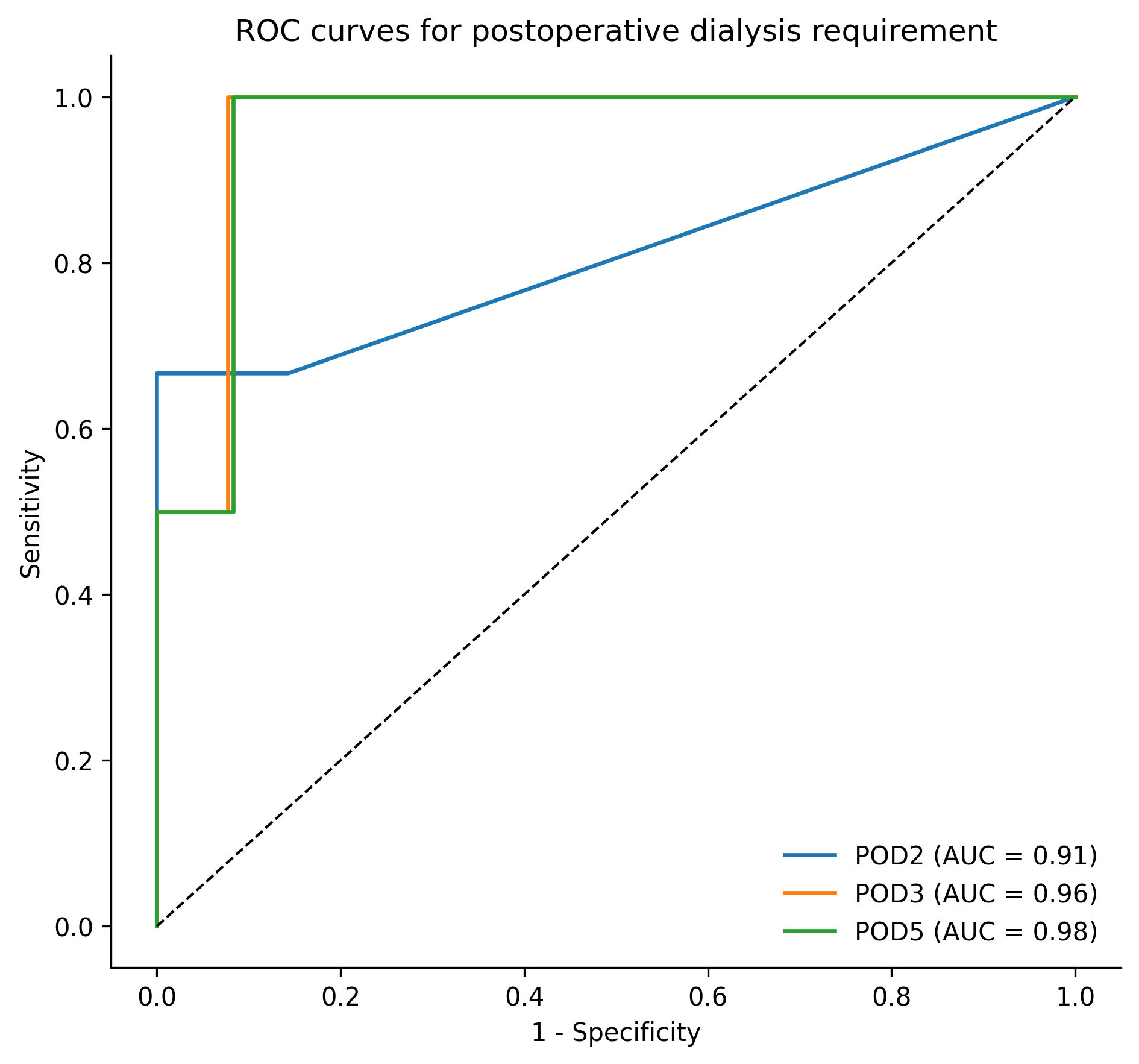


***Supplementary Fig. 2*** *Receiver operating characteristic curves for bioactive adrenomedullin (bioADM) in predicting postoperative dialysis requirement at POD2, POD3, and POD5.*

| **Outcome/ Time point** | **Analysis** | **Estimate** |
| --- | --- | --- |
| **Vasopressor requirement** | | |
| Postoperative course | Group comparison | p = 0.0356 |
| **AKIN** | | |
| POD2 | Group comparison | Mann–Whitney p = 0.019 |
| POD2 | Discrimination | AUC 0.714 (95% CI, 0.389–0.976) |
| **AKIN severity** | | |
| Baseline | Association | rho = 0.657 (p = 0.004) |
| POD2 | Association | rho = 0.691 (p = 0.004) |
| POD3 | Group comparison | Mann–Whitney p = 0.084 |
| POD3 | Association | rho = 0.452 (p = 0.096) |
| **Dialysis requirement** | | |
| POD2 | Discrimination | AUC 0.91 (95% CI, 0.73–1.00) |
| POD3 | Discrimination | AUC 0.96 (95% CI, 0.88–1.00) |
| POD5 | Discrimination | AUC 0.98 (95% CI, 0.92–1.00) |

***Supplementary Table 1*** *Detailed statistical results for organ-specific exploratory analyses.*

| **Outcome** | **Analysis** | **Estimate** |
| --- | --- | --- |
| Major Complications | Discrimination | AUC 0.562 (95% CI, 0.247–0.851) |
| Major Complications | Logistic regression | OR 1.19 (95% CI, 0.24–5.88); p = 0.833 |
| Clavien-Dindo grade | Association | rho = 0.027 (p = 0.917) |
| 30-day CCI | Association | rho = − 0.060 (p = 0.828) |

***Supplementary Table 2*** *Detailed statistical results for overall postoperative morbidity analyses.*

**Abbreviations:** AKIN, acute kidney injury; AUC, area under the curve; bioADM, bioactive adrenomedullin; CCI, Comprehensive Complication Index; CI, confidence interval; POD, postoperative day.
